# Mr. Palmer's Case of Hydrocephalus Internus

**Published:** 1805-09-01

**Authors:** Lionel Palmer

**Affiliations:** Mattishall


### *To the Editors of the Medical and Physical Journal.*

Gentlemen,

If you judge the following case merits a place in your excellent Journal, you will have the goodness to insert it. On Friday, July the 12th ult. I was called to attend S. L. a remarkable, active, intelligent child, three years and a half old; who on Monday the 8th preceding was suddenly indisposed with pain in his head, attended with vomiting, and purging, of a green and bilious matter, which ceased at night, and was succeeded by obstinate costiveness; the pain in his head continued, accompanied with fever and delirium to an alarming degree, the two preceding nights and days prior to my being called in, which left him extremely dull and heavy, with interrupted sleep, and a continual melancholic whine, with alternate general and partial flushing. His pulses were quick, small, and intermitting. His urine very scanty, not a small tea cup full in twenty-four hours. He constantly laid in one position with his right arm over his eyes, and with his left he continually kept feeling on the centre of the left parietal bone. His thirst was considerable, but he refused every kind of drink except water. From the beginning to the end of his indisposition he had no appetite; and added to the above symptoms, he had a slight cough some days previous to his being indisposed, which became so violent as to threaten suffocation. Judging the case to be hydrocephalus internus, calomel was prescribed in sufficient doses to evacuate the bowels, with a fever mixture, and blisters to his head and neck for three days, without any relief. On the 16th, lifting up his arm, and examining his eyes, I found both the pupils very much dilated and unirritable. He could not bear to have his head lifted from the pillow, or to be turned in the bed; on which I concluded it a lost case, and informed the parents I considered it as such; but they, being extremely anxious for the child's life, made me determine to adopt some other mode of treatment, with a view if possible to accomplish a cure. I accordingly made trial of digitalis; (but for the hint I must acknowledge myself indebted to the writings of that eminent philosopher and physician, the late Dr. E. Darwin, whose saturated tincture I used, with the compound tincture of lavender, to cover in some measure its disagreeable taste); taste); the following is the formula I tried: Tinct. lavend. comp. tinct. digitalis aa. ʒij. M. Wishing to have the constitution under the influence of the digitalis as soon as possible, ten drops were given three times a day, in a glass of water, increasing three or four drops each dose every day, until twenty-four were taken ter in die. But as the digitalis affected the bowels by producing too many motions; fearing it might debilitate too much, I thought proper to restrain the frequency of that operation; and for this purpose, prescribed the following drops: Tinct. opii camphorat. ʒiijß. tinct. opii ʒß. M. cap. coch. j. parv. omni nocte, which had the desired effect. No visible alteration for the better was perceived till the 19th, (the fourth day of using the drops) when the pupils of the eyes were less dilated and became irritable; the cough very much abated; the urine more plentiful with a copious perspiration; the pulse slower and uninterrupted; sleep refreshing. My patient now became cheerful, called for his *play-things*, and toasted bread buttered, which he ate. The digitalis was continued till the 22d day, when every former symptom vanished; and as only debility remained, a bark mixture with the night drops were given four or five days and nights longer, which completed the cure.

This case I have faithfully described, and as accurately as I possibly could, with an anxious wish, that, for the benefit of the little sufferers who labour under this obstinate, and generally fatal disease, and for the comfort and happiness of their parents, a more extensive trial may be given to digitalis, with a cautious but spirited hand; as it seems likely to prove a very efficacious remedy in hydrocephalus ex et internus, when not arising from any mechanical obstruction of the brain. I think the modus operandi of digitalis bespeaks its efficacy, for as it powerfully retards the circulation of the blood, it thereby prevents the hydrocephalic fluid from increasing on the brain; and which also gives time for the absorption of what may be already accumulated; while the nausea, which it seldom fails to keep up in well regulated doses, oftentimes produces a copious diaphoresis; and it always causes alvine and urinary evacuations. It evidently acted in this case as a sudorific, cathartic, and diuretic, and in my opinion, by that means so effectually and speedily carried off the disease. It could not be the blisters, as they did not vesicate; nor the calomel, as its effects produced no alteration. My practice being considerably among children, I intend to make farther trials with the digitalis as cases may occur, the the result of which, (if this be accepted) I will communicate for general good; fervently hoping it may stimulate others to do the same. I am, &c.

*Mattishall, Aug.* 7, 1805. LIONEL PALMER.

